# *Tv*-RIO1 – an atypical protein kinase from the parasitic nematode *Trichostrongylus vitrinus*

**DOI:** 10.1186/1756-3305-1-34

**Published:** 2008-09-22

**Authors:** Min Hu, Nicole LaRonde-LeBlanc, Paul W Sternberg, Robin B Gasser

**Affiliations:** 1 Department of Veterinary Science, The University of Melbourne, 250 Princes Highway, Werribee, Victoria 3030, Australia; 2 Department of Chemistry and Biochemistry, Center for Biomolecular Structure and Organization, University of Maryland, College Park, MD 20742-3360, USA; 3 Biology Division, California Institute of Technology, Pasadena, CA 91125, USA

## Abstract

**Background:**

Protein kinases are key enzymes that regulate a wide range of cellular processes, including cell-cycle progression, transcription, DNA replication and metabolic functions. These enzymes catalyse the transfer of phosphates to serine, threonine and tyrosine residues, thus playing functional roles in reversible protein phosphorylation. There are two main groups, namely eukaryotic protein kinases (ePKs) and atypical protein kinases (aPKs); RIO kinases belong to the latter group. While there is some information about RIO kinases and their roles in animals, nothing is known about them in parasites. This is the first study to characterise a RIO1 kinase from any parasite.

**Results:**

A full-length cDNA (*Tv-rio-1*) encoding a RIO1 protein kinase (*Tv*-RIO1) was isolated from the economically important parasitic nematode *Trichostrongylus vitrinus *(Order Strongylida). The uninterrupted open reading frame (ORF) of 1476 nucleotides encoded a protein of 491 amino acids, containing the characteristic RIO1 motif LVHADLSEYNTL. *Tv-rio*-1 was transcribed at the highest level in the third-stage larva (L3), and a higher level in adult females than in males. Comparison with homologues from other organisms showed that protein *Tv*-RIO1 had significant homology to related proteins from a range of metazoans and plants. Amino acid sequence identity was most pronounced in the ATP-binding motif, active site and metal binding loop. Phylogenetic analyses of selected amino acid sequence data revealed *Tv*-RIO1 to be most closely related to the proteins in the species of *Caenorhabditis*. A structural model of *Tv*-RIO1 was constructed and compared with the published crystal structure of RIO1 of *Archaeoglobus fulgidus (Af*-Rio1).

**Conclusion:**

This study provides the first insights into the RIO1 protein kinases of nematodes, and a foundation for further investigations into the biochemical and functional roles of this molecule in biological processes in parasitic nematodes.

## Background

Protein kinases are a group of enzymes essential for the regulation of a large variety of cellular processes, including cell-cycle progression, transcription, DNA replication and metabolic functions [1]. These enzymes catalyse the transfer of phosphates to serine, threonine and tyrosine residues, thus playing functional roles in reversible protein phosphorylation. In organisms, such as *Homo sapiens*, *Mus musculus*, *Drosophila melanogaster *(vinegar fly), *Caenorhabditis elegans *(worm), *Saccharomyces cerevisiae *(yeast), *Dictyostelium discoideum *(slime mould) and *Plasmodium falciparum *(malaria parasite), the complete complement of protein kinases has been identified *via *the analysis of genome sequences [2]. Based on their structure, protein kinases can be classified into two main groups, namely eukaryotic protein kinases (ePKs) and atypical protein kinases (aPKs) [3]. The ePKs usually have 11 subdomains, including a nucleotide-binding loop (subdomain I), typically with the sequence GXGXXG, which binds and orients the phosphates of ATP; a hinge region which interacts with the adenine moiety of the ATP; a catalytic loop (subdomain VIb) which contains conserved catalytic Asn and Asp residues involved in phosphoryl transfer; a metal-binding or "DFG" loop (subdomain VII) for the positioning of metal ions; and an activation loop (subdomain VIII) [4]. The aPKs are enzymes with protein kinase activity and limited sequence similarity to any known ePKs. Of the 518 kinases known to be encoded in the human genome, 40 have been identified as aPKs, which have been classified into 13 families or groups, one of which represents the RIO kinases [3]. These serine kinases are conserved in sequence among a range of different organisms, yet are quite divergent from kinases of other families with known structures [5,6].

Through sequence and structural analyses, RIO proteins have been found to contain the conserved signature residues, typifying protein kinases [7,8]. RIO kinases are present in organisms from archaea to humans, suggesting key fundamental roles in the Metazoa. The function of RIO1 was first investigated in yeast [9]. It was found that RIO1 is a non-ribosomal protein located in the cytoplasm and specifically required for 20S precursor ribosomal RNA (pre-rRNA) processing; the depletion of the protein RIO1 caused the inhibition of 18S rRNA production and an accumulation of the 20S pre-rRNA in the cytoplasm. Further sequence characterisation of RIO1 of *Saccharomyces cerevisiae *indicated that this protein was a serine kinase [8]. Although the primary sequences of RIO1 proteins are quite divergent from those of members of other protein kinase families, their structural folding is similar to known canonical protein kinases. Also, they display protein kinase activity *in vitro*. Analysis by mutagenesis [8] showed that some of the conserved residues are crucial for enzymatic activity, and cytological study of RIO1 has revealed that it also plays an important role in cell-cycle progression (in G1 to S transition and in the control of the onset of anaphase) as well as the maintenance of mitotic chromosome stability [8]. In contrast to RIO1, RIO2 kinase appears to be localized predominantly to the nucleus [10]. The biological activities of these two proteins neither complement each other nor do they co-purify, although both are associated with the same fractions containing 20S precursor RNA in yeast [11].

Although not yet studied in detail in multicellular organisms, RIO1 kinases are encoded in the genome of the free-living nematode *Caenorhabditis elegans *[12]. RNA interference (RNAi), which decreases messenger RNA (mRNA) levels of the targeted *C. elegans *gene, has been shown to affect predominantly embryonic and larval growth and/or development [13-17]. In spite of the functional importance of this molecule, there is no published information regarding its structure or function for any parasitic nematode. Clearly, the isolation and characterisation of RIO protein kinases from nematodes would provide a starting point for a better understanding of their roles in developmental processes. In a genomic study [18], we characterised an expressed sequence tag (EST), designated TVf09_H06, from an adult female-enriched cDNA library for *Trichostrongylus vitrinus*, an economically important parasitic nematode. The amino acid sequence inferred from this EST had the highest homology (e-value: 2e^-37^) to the protein encoded by predicted RIO1 kinase (M01B12.5; see ) from *C. elegans *(unpublished findings). Therefore, the aims of this study were to isolate and characterise the full-length complementary DNA (cDNA) of RIO1 of *T*.*vitrinus *corresponding to TVf09_H06 and carry out comparative analyses with related molecules encoded by other organisms, to explore transcription in different developmental stages and to predict its three-dimensional structure by comparison with known crystal structures of homologues.

## Methods

### Parasite propagation

Merino lambs (males; 8–12 weeks of age), maintained under helminth-free conditions, were inoculated intra-ruminally with 30,000 infective third-stage larvae (L_3_) of *T. vitrinus*. The patency of the infection (~24 days after inoculation) was established by the detection of strongylid eggs in the faeces using the McMaster flotation method [19]. First- and second-stage larvae (L1 and L2) and L3 were collected after 1, 3 and 7 days of incubation of faeces at 28°C, respectively, and purified by repeated sedimentation and a migration through a nylon sieve (mesh size: 20 μm) for 16 h. For the collection of fourth-stage larvae (L_4_) and adults of *T*. *vitrinus*, infected lambs were euthanized by intravenous administration of an overdose of pentobarbitone sodium (Lethobarb, Virbac Pty. Ltd.) 8 and 30 days after intra-ruminal inoculation, respectively. Adult worms were collected (using fine forceps) from the chyme from the first 4 m of the small intestine, washed extensively in chilled (4°C) phosphate-buffered saline (PBS), and males and females (adults) separated prior to snap freezing in liquid nitrogen and subsequent storage at -70°C.

### Isolation, purification, treatment and storage of nucleic acids

Total genomic DNA was extracted from ~0.5 g of single sex (male or female) adult worms using a small-scale sodium dodecyl-sulphate (SDS)/proteinase K extraction procedure [20], followed by mini-column (Wizard^® ^Clean-Up, Promega) purification. The specific identity of the parasite material in each sample was confirmed by PCR amplification of the second internal transcribed spacer (ITS-2) of nuclear ribosomal DNA from genomic DNA and subsequent, automated sequencing [20,21]. The sequences determined were required to be identical to the ITS-2 sequence with GenBank accession no. X78064[21].

Total RNA was extracted separately from different developmental stages (L1, L2, L3, L4 or adults) or sexes of *T. vitrinus *(homogenized under liquid nitrogen using a mortar and pestle) employing the TriPure isolation reagent^® ^(Roche Molecular Biochemicals). RNA yields were estimated spectrophotometrically (ND-1000 UV-VIS spectrophotometer, v.3.2.1, NanoDrop Technologies), and the integrity of RNA was confirmed *via *the detection of discrete 18S and 28S ribosomal RNA bands on ethidium bromide-stained gels. Each RNA sample (~10 μg) was treated with 2 U of *DNase *I (Promega) and incubated at 37°C for 30 min prior to heat denaturation of the enzyme (75°C for 5 min). Both DNA and RNA samples were stored at -70°C.

### Isolation of the full-length cDNA encoding RIO1 kinase from *T. vitrinus*

Using four gene-specific primers PK1F (forward: 5'-CGACTTCTCCAGCGTGGAACGTTAACC-3'); PK2R (reverse: 5'-GGAACCAGGGAGACCCGCTTGATGCT-3'); PK3F (forward: 5'-GAACCGGCTACTGCAAACACAACCCCCG-3'); PK4R (reverse: 5'-GCGGTGCACCCCAGCCATCACGACC-3') designed to the sequence of EST TVf09_H06 (accession no. NP_491102.2), two partially overlapping cDNA fragments were produced separately from total RNA from adult female worms using 5'- and 3'-rapid amplification of cDNA ends (RACE) (SMART™ RACE cDNA Amplification Kit, BD Biosciences). The cDNAs were ligated separately into the pGEM-T-Easy^® ^vector (Promega); *Escherichia coli *(strain JM109) (10^8 ^colony forming units/μg) was transformed with recombinant plasmids *via *heat shock and then grown overnight at 37°C on Luria Bertani (LB) plates containing 10 mg/ml ampicillin, 0.5 mM isopropyl-β-d-thiogalactopyranoside (IPTG) and 80 μg/ml X-gal (5-bromo-4-chloro-3-indolyl-β-galactosidase). Plasmid DNA was isolated from recombinant clones and column-purified (Wizard^®^, Promega) from overnight cultures, and inserts sequenced in both directions using vector oligonucleotide primers (M13 and SP6), employing Big Dye Terminator v.3.1 chemistry in an automated ABI-PRISM sequencer (Applied Biosystems). Based on the resultant sequences, selected oligonucleotide primers were designed to amplify the full-length *Tv-rio-1 *cDNA from *T. vitrinus *adult females, which was subsequently cloned and sequenced.

### Bioinformatic analyses

Nucleotide sequences were assembled using the program EGassembler [22]. The full-length cDNA sequence of *Tv*-*rio-1 *was conceptually translated (six different frames) into amino acid sequences using the Baylor College of Medicine (BCM) Search Launcher [23] and aligned using the program ClustalW [24]. Sequences were compared with those available in public, non-redundant databases using BLASTn and BLASTx algorithms [25] available *via *the National Center for Biotechnology Information [26], the Sanger Centre [27] and the Parasite Genome database [28], in order to verify the identity of the molecules isolated. Protein motifs were identified by scanning the database PROSITE [29] and Pfam [30].

Phylogenetic analyses of inferred primary amino acid sequence data were conducted using the program PAUP*4.0b10 [31], as described previously [32]. In brief, the neighbour-joining (NJ) method was used to construct trees from distance data. The maximum parsimony (MP) method, based on character state analysis, was also used. Characters were treated as unordered and were weighted equally; alignment gaps were treated as "missing" in all analysis. Exhaustive searches with tree-bisection-reconnection (TBR) branch swapping were used to infer the shortest trees. The length, consistency index, excluding uninformative characters, and the retention indices of each most parsimonious tree were recorded. Bootstrap analyses (1000 replicates) were conducted using heuristic searches and TBR branch swapping, with the MulTrees option, to determine the relative support for clades in the consensus trees.

The three-dimensional structure of the RIO1 kinase of *Archaeoglobus fulgidus *(*Af*-RIO1 kinase; PDB code 1ZTF; *cf. *[5,6]) was used to create an homology model of the RIO domain of *Tv*-RIO1 using the program DeepView Swiss-PdbViewer and the SWISS-MODEL server [33]. The energy of the resultant model was minimized in DeepView using Gromos 96 [33]. The model did not include the C-terminal extension, characteristic of sequences of eukaryotic RIO1 proteins, including *Tv*-RIO1.

All comparative analyses with *C. elegans *were carried out using WormBase [34]. Genetic interactions with the *C. elegans *orthologue (gene name M01B12.5) of *Tv*-RIO1 were predicted using the probabilistic functional gene network for *C. elegans *(see [35]); this computational network covers the majority (82%) of *C. elegans *genes and makes predictions regarding essentiality, spanning diverse cellular and developmental processes [35].

### Reverse transcription real-time polymerase chain reaction (PCR)

Double-stranded cDNA was synthesized from total RNA separately from each stage and sex of *T. vitrinus *using reverse transcriptase (Superscript III, Invitrogen). Briefly, 5 μg of total RNA were added to 14 μl of H_2_O and 1 μl of oligo d(T)n = 12–18 primer (0.5 μg/μl), heated to 70°C for 10 min and chilled on ice. First- and second-strand cDNAs were synthesized *via *the addition of 4 μl of first-strand cDNA buffer (250 mM Tris-HCl, pH 8.3, 375 mM KCl and 15 mM MgCl_2_), 2 μl of 0.1 M dithiothreitol, and 1 μl of 10 mM of each dNTP, followed by an incubation at 25°C for 10 min, 42°C for 50 min and 70°C for 15 min. The transcripts (176 bp) representing *Tv-rio-1 *were amplified from individual cDNAs by real-time PCR using the primer pair PK3F-PK4R. The PCR amplification of a portion (187 bp) of the large subunit (28S) ribosomal RNA using primers 28S1/F (5'-GCATTAGCTCTCGCGTTACC-3') and 28S3/R (5'-GAGAGGGACAGCAGGTTCAC-3'), previously determined to be present equally in each developmental stage and sex in a related parasitic nematode, *Oesophagostomum dentatum *(see [36]), was used as a positive control. Samples without template (no-DNA controls) were included in each PCR run. For each sample, ~0.5 μg of cDNA was subjected to PCR (20 μl) using the SYBR^® ^GreenER™ qPCR SuperMix Universal (Cat. no. 11762-100, Invitrogen) in a Rotor-Gene™ 3000 thermal cycler (Corbett Research) under the following conditions: one cycle of 50°C for 5 min and of 95°C for 10 min, followed by 40 cycles of 95°C for 15 s, 60°C for 30 s and 72°C for 30 s. Each sample was tested in triplicate, using a calibrator (28S) as well as positive and no-template controls. The specificity and identity of individual amplicons were verified by melting curve analysis and subsequent direct, automated sequencing using the same primers (individually) as employed for the PCR. Relative transcriptional differences were calculated from normalised values using method described by Livak and Schmittgen [37].

## Results and discussion

### Characterisation of *Tv*-RIO1 and relationship with selected homologues

**Figure 1:**
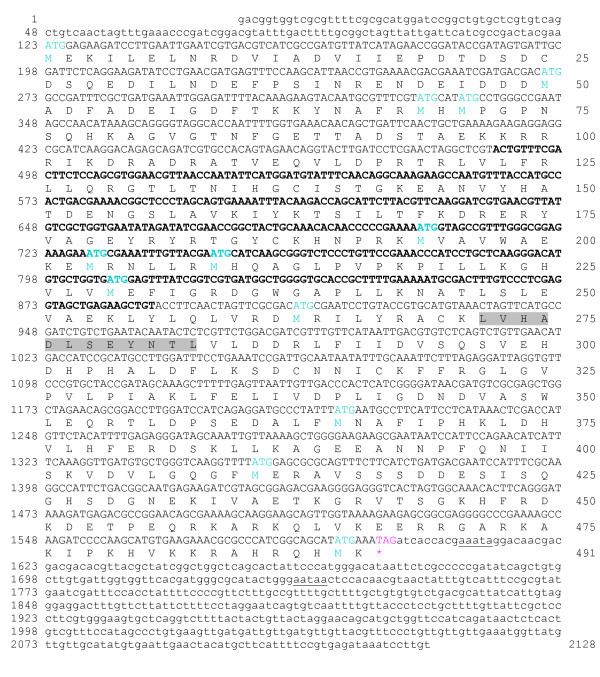
**Full-length cDNA sequence of the cDNA (*Tv-rio-1*) encoding the protein *Tv*-RIO1 from *Trichostrongylus vitrinus*, and its predicted amino acid sequence**. The nucleotide sequence determined from the original EST TVf09_H06 (accession no. NP_491102.2; [18]) is in bold text. The untranslated regions are in lower case, and protein-coding nucleotide sequence is in upper case. ATG and TAG (asterisk) are the inferred translation initiation and termination signals, respectively; the putative polyadenylation signal sequence is underlined. The amino acid residues representing the RIO1 protein kinase signature LVHADLSEYNTL (PS01245) are shaded.

**Figure 2:**
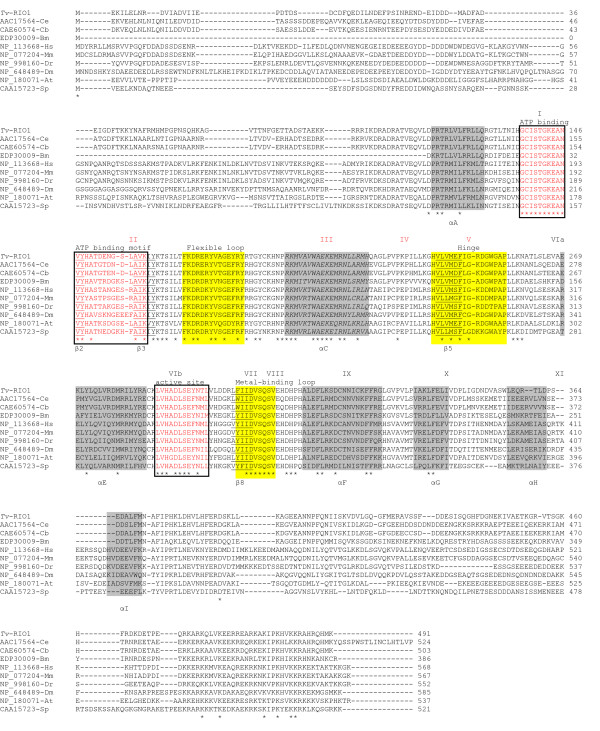
**An alignment of the inferred amino acid sequences of RIO1 proteins from *Trichostrongylus vitrinus *and other nine selected species**. These nine species include *Caenorhabditis elegans *(accession no. AAC17564), *C. briggsae *(CAE60574), *Brugia malayi *(EDP30009), *Homo sapiens *(NP_113668), *Mus musculus *(NP_077204), *Danio rerio *(NP_998160), *Drosophila melanogaster *(NP_648489), *Arabidopsis thaliana *(NP_180071) and *Schizosaccharomyces pombe *(CAA15723). Amino acids predicted to be involved in the ATP binding motif and active sites are boxed. Identical amino acids are marked with asterisks. Predicted subdomains I-XI (see Fig. 1B; [7]) are marked above the alignment. Alpha-helices A-I (shaded) or beta-sheet structures (underlined) [1] are indicated. Flexible loop, hinge and metal-binding loop were identified (see Fig. 1D in [40]). Dashes indicate gaps in the sequence, included for alignment purposes.

The full-length cDNA (designated *Tv-rio-1*) isolated by RACE was 2128 nucleotides (nt) (GenBank accession no. FM209038; Fig. 1) in size and contained a 5'-UTR of 122 nt, an open reading frame (ORF) of 1476 nt and 3'-UTR of 530 nt. A putative polyadenylation signal (AATAA) was identified and located 111 nt 3' of the stop codon. The conceptually translated protein was 491 amino acids in length and its sequence contained the signature LVHADLSEYNTL (PS01245) characteristic of the RIO1 kinase family [8]. Comparisons with sequences in non-redundant databases conducted by BLASTx analysis showed that *Tv*-RIO1 has significant similarities to related sequences from a range of organisms, including other nematodes, insects, vertebrates and plants, although the vast majority of them have yet to be characterised. The highest amino acid similarities recorded were to the proteins inferred from *C. briggsae *gene CBG4203 (e-value: 1e^-150^; 60% of identity and 74% of similarity) and from *C. elegans *gene M01B12.5 (e-value: 1e^-150^; 60% of identity and 73% of similarity) (see ), now recognized as the RIO1 protein kinase. Pairwise comparisons of amino acid sequence differences between *Tv*-RIO1 and selected sequences representing other phyla (mouse, human, vinegar fly, zebra-fish and yeast; Table 1) revealed sequence identities ranging from 22.3% to 64.5%. An alignment of the amino acid sequences of selected RIO1 kinases (Fig. 2) showed conserved regions in the ATP-binding motif (subdomains I and II) and the active site (subdomain VIb) and other subdomains, including III, V, VII, VIII, IX and X, suggesting that *Tv*-RIO1 is functionally similar to other RIO1 kinases (*cf. *[8]). The alignment also showed that the amino acid sequences in regions external to these subdomains were more divergent (Fig. 2) than the sequences in the subdomains.

**Table 1:** Pairwise comparison of amino acid identity (%) among RIO1 protein kinase of *Trichostrongylus vitrinus *and other selected species for which full-length cDNA sequences encoding RIO1 protein kinases are predicted from publicly available data

	***Tv***	***Ce***	***Cb***	***Bm***	***At***	***Dm***	***Dr***	***Hs***	***Mm***	***Sp***
***Tv***	-									
***Ce***	65.2	-								
***Cb***	64.5	93.7	-							
***Bm***	43.4	45.3	44.4	-						
***At***	32.6	29.3	25.9	22.9	-					
***Dm***	32.1	32.1	31.0	27.6	36.3	-				
***Dr***	31.0	32.6	32.6	25.3	45.3	48.2	-			
***Hs***	27.6	31.0	30.4	27.6	44.4	49.6	79.4	-		
***Mm***	25.9	29.3	27.6	25.3	40.4	47.2	73.9	87.6	-	
***Sp***	22.3	23.5	21.7	13.5	19.9	33.7	28.7	30.4	28.2	-

*Abbreviations*: *Tv*, *Trichostrongylus vitrinus*; *Ce, Caenorhabditis elegans*; *Cb*, *C. briggsae*; *Bm, Brugia malayi*; *At, Arabidopsis thaliana*; *Dm, Drosophila melanogaster*; *Dr, Danio rerio*; *Hs, Homo sapiens*; *Mm, Mus musculus*; *Sp, Schizosaccharomyces pombe*; refer to Fig. 2 for accession numbers

**Figure 3:**
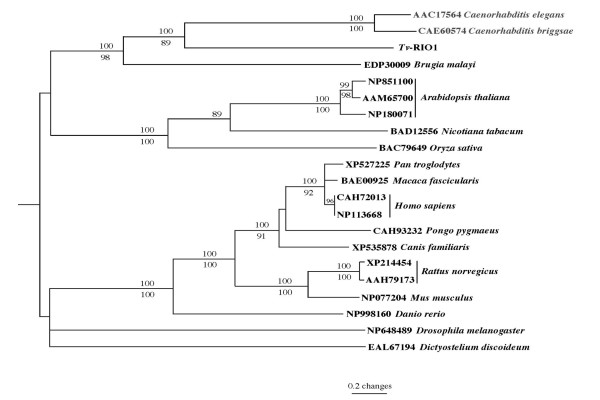
**Genetic relationship of *Tv*-RIO1 with homologues from a range of organisms**. Neighbour-joining tree displays the relationship of *Tv*-RIO1 from *Trichostrongylus vitrinus *with RIO1 protein kinases from 16 species representing different phyla. These species are *Caenorhabditis elegans*, *C. briggsae*, *Brugia malayi *(nematodes); *Arabidopsis thaliana*, *Nicotiana tabacum*, *Oryza sativa *(plants); *Homo sapiens*, *Macaca fascicularis*, *Pan troglodytes*, *Pongo pygmaeus *(primates); *Canis familiaris*, *Rattus norvegicus*, *Mus musculus *(other mammals); *Danio rerio *(zebrafish); *Drosophila melanogaster *(vinegar fly) and *Dictyostelium discoideum *(slime mould). Accession numbers identify individual sequences representing individual species. *Schizosaccharomyces pombe *(fission yeast) (accession no. CAA15723) represents the outgroup. Numbers above and below the branches are the bootstrap values (%) obtained using the neighbour-joining and maximum parsimony methods, respectively.

The full-length amino acid sequences of *Tv*-RIO1 (inferred from cDNA *Tv-rio-1*) and 21 other homologues representing a range of different species were aligned and subjected to phylogenetic analyses (Fig. 3). There was concordance in topology between the MP and NJ trees, and *Tv*-RIO1 was closely related to *Ce*-RIO1 and *Cb*-RIO1. The four RIO1 kinases from these nematodes clustered together, with strong bootstrap support (98–100%), to the exclusion of molecules from organisms from other phyla; molecules from plants and vertebrates each formed separate clades, also supported by strong bootstrap values (Fig. 3).

### Structural model for *Tv*-RIO1

Recently, crystal structures of the proteins RIO1 and RIO2 have been characterised from *Archaeoglobus fulgidus *[5,6], allowing RIO kinases to be defined as a distinct, novel family of protein kinases. The structure of RIO2 was first investigated, revealing the presence of two domains [38]. The N-terminal domain, conserved for the RIO2 family members and not present in the RIO1 protein kinase family, is structurally homologous to the winged helix (wHTH) domain, seen primarily in DNA-binding proteins. The C-terminal domain, the sequence of which is conserved between both proteins RIO1 and RIO2, is structurally homologous to known protein kinase (ePK) domains. The ePKs usually contain 11 conserved subdomains that form the catalytic core. Compared with ePKs, RIO kinase domains resemble a trimmed version of an ePK kinase domain, containing only eight of these subdomains, lacking the activation loop (subdomain VIII) and subdomains X and XI which are important for substrate binding [38]. Detailed study of RIO1 and comparisons with RIO2 [39,40] have defined the minimal consensus RIO-domain that, like ePKs, also contains an N-lobe, a hinge region, and a C-lobe. However, the RIO kinase domain contains only three α-helices (αE, αF and αI) in the C-lobe. All RIO domains contain an additional α-helix (αR) N-terminal to the canonical N-lobe and a loop inserted between αC and β3 called "flexible loop". Study of nucleotide binding by the RIO kinases and comparison with canonical protein kinases [5,6] has identified specific phosphate-binding loops for subfamily RIO1 domains (with the sequence STGKEA) and RIO2 domains (with the sequence GxGKES), which are significantly different from their counterpart in ePKs. This finding indicates that the interaction of RIO kinases with the phosphate moiety and protein substrate is different from ePKs.

**Figure 4:**
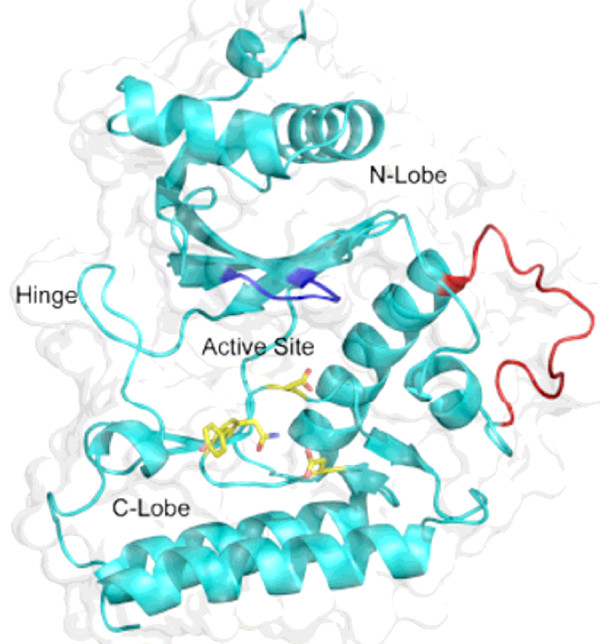
**Homology model of the *Tv*-RIO1 protein from *Trichostrongylus vitrinus***. The loop in red indicates the region of dissimilarity between *Tv*-RIO1 and *Af*-RIO1 from *Archaeoglobus fulgidus*. The P-loop required for the binding to phosphate is shown in blue. Catalytic residues (Asn 281, Asp 276 and Asp 293) and the highly conserved Tyr 280 are indicated by yellow sticks.

Comparison between protein sequences *Tv*-RIO1 and *Af*-RIO1 (from *A. fulgidus*) revealed 38% identity in the ~240 residues containing the RIO domain of *Tv*-RIO1 (92–325). Based on the crystal structure of *Af*-RIO1 [38], the homology model constructed for *Tv*-RIO1 (Fig. 4) confirmed that differences between the eukaryotic homologue and the archaeal RIO1 lie mainly in loop regions which do not contribute to the packing of the three dimensional structure of the molecule. Most notable is the loop between the third beta-strand and the alpha-helix C of the kinase domain (Fig. 4). This loop, being highly conserved among eukaryotes, is longer in *Tv*-RIO1 compared with that in *Af*-RIO1, and is highly likely to adopt a different conformation. The residue identified as the auto-phosphorylation site in this loop in *Af*-RIO1 is absent from *Tv*-RIO1 and RIO1 proteins from other eukaryotes; therefore, autophosphorylation is likely to occur at an alternate site. Within this loop in *Tv*-RIO1, there is only one available serine, Ser 165, which in the homology model is positioned at the end of beta-strand 3. The auto-phosphorylation site identified for RIO2 from *A. fulgidus *also lies at the end of this strand, suggesting that such an autophosphorylation site is indeed possible for eukaryotic RIO1 proteins. *Tv*-RIO1 also contains an extended conserved lysine-rich, C-terminal domain present in all RIO1 proteins of eukaryotes but not that of the archaeal *A. fulgidus *(see [40]). It is presumed that this region is required for the regulation of RIO1 proteins in eukaryotes. A recent report [41] has indicated that the C-terminal region of RIO1 is required for the interaction with and phosphorylation by CK2 in yeast (*S. cerevisiae*), and that this phosphorylation regulates the degradation of RIO1 at the G1/S transition in the cell-cycle.

### Transcription in different developmental stages and prediction of function based on comparisons with *C. elegans*

**Figure 5:**
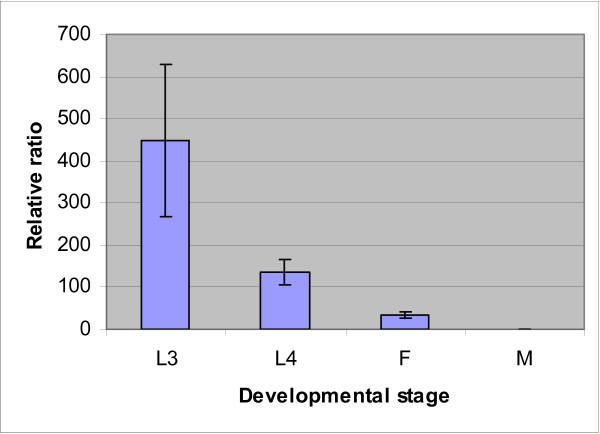
**Transcription of *Tv-rio-1 *in *Trichostrongylus vitrinus***. Transcriptional profile of *Tv-rio*-1 in different developmental stages [third – (L3) and fourth – (L4) stage larvae] and genders [females (F) and males (M)] of *Trichostrongylus vitrinus*, determined by real-time PCR analysis. Data shown are mean values (± standard error of the mean) derived from three replicates in repeat experiments. Relative transcription was calculated by normalization of the raw data, followed by the determination of abundance relative to a calibrator. Quantification of the cDNA representing *Tv*-*rio1 *in each sample was normalised, using part of the large subunit (28S) of the nuclear ribosomal RNA gene as an endogenous control.

**Figure 6:**
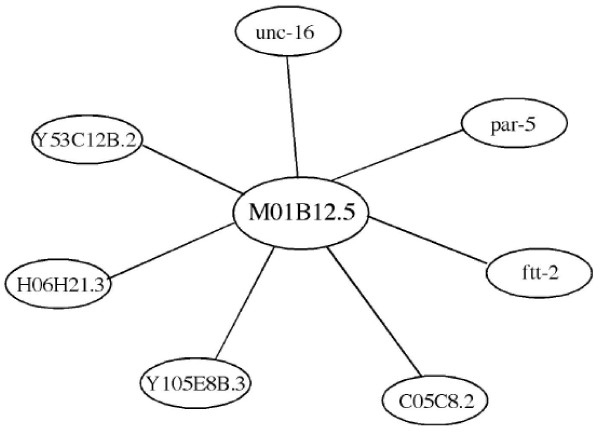
**Probabilistic functional gene network analysis for *Caenorhabditis elegans *gene M01B12.5**. (using the recommended cut-off value; [35]).

Real-time PCR analysis (Fig. 5) showed that the transcription of *Tv-rio-1 *was greatest in third-stage larvae (L_3_) of *T. vitrinus*. The level of transcription of *Tv*-*rio-1 *decreased from the L_3 _to the adult worm (Fig. 5), and transcription was significantly greater (~33 times) in the adult female compared with the male of *T. vitrinus*. In spite of the limited information on the transcription and function of RIO1 protein kinases for most multicellular organisms, there are important unpublished data for the free-living nematode *C. elegans*. In this nematode, genome-wide RNAi experiments have revealed 'loss-of-function' phenotypes for the gene M01B12.5; these included embryonic lethality (Emb), slow growth (Gro), larval arrestment (Lva), larval lethal (Lvl), sick larvae (Sck), fat content reduced, and reduced brood size (*cf. *[13-17]). Gene ontology (GO) analysis also indicates that RIO1 kinase plays roles in embryonic development, larval development, positive regulation of growth rate and multicellular organism growth in *C. elegans *(see WormBase), suggesting its overall functional importance in growth and development of nematodes. Probabilistic functional gene network analyses [33] predicted that the *C. elegans *gene M01B12.5 interacts with seven other molecules, including Y53C12B.2, C05C8.2, Y105E8B.3, H06H21.3, *unc-16*, *par-5 *and *ftt-2 *(Fig. 6). Among them, homologues/orthologues of Y53C12B.2, C05C8.2 and H06H21.3 from other species are involved in RNA binding or nuclear ribosomal RNA processing, suggesting that M01B12.5 may also be involved in ribosomal RNA biosynthesis. Although detailed studies have not yet been conducted, the availability of gene silencing and transgenesis in *C. elegans *provides excellent scope for detailed investigations into the functional roles of gene M01B12.5 and the RIO1 protein kinase it encodes.

## Conclusion

This study characterised an atypical protein kinase – RIO1 protein kinase – from an economically important parasitic nematode, *Trichostrongylus vitrinus*. The findings from the study provide the first insights into the RIO1 protein kinases of nematodes, and a foundation for further investigations into the biochemical and functional roles of this molecule in biological processes in parasitic nematodes.

## Competing interests

The authors declare that they have no competing interests.

## Authors' contributions

RBG and MH conceived the project, collected the parasite material, analysed the data and drafted the manuscript. MH carried out the molecular work. NLL and PWS assisted in analyses, interpretation and the drafting of the manuscript. All authors read and approved the final manuscript.
